# Metallophore Activity toward the Rare Earth Elements by Bacteria Isolated from Acid Mine Drainage Due to Coal Mining

**DOI:** 10.3390/microorganisms11112672

**Published:** 2023-10-31

**Authors:** Stephanie Skeba, Morgan Snyder, Chris Maltman

**Affiliations:** Department of Biology, Slippery Rock University, Slippery Rock, PA 16057, USA

**Keywords:** rare earth elements, metallophore, siderophore, acid mine drainage, coal mining

## Abstract

The field of microbe–metal interactions has been gaining significant attention. While the direct impact of metal oxyanions on bacteria has been investigated, significantly less attention has been placed on the ability of certain microbes to ‘collect’ such metal ions via secreted proteins. Many bacteria possess low-weight molecules called siderophores, which collect Fe from the environment to be brought back to the cell. However, some appear to have additional roles, including binding other metals, termed ‘metallophores’. Microbes can remove/sequester these from their surroundings, but the breadth of those that can be removed is still unknown. Using the Chromeazurol S assay, we identified eight isolates, most belonging to the genus *Pseudomonas*, possessing siderophore activity, mainly from sites impacted by coal mine drainage, also possessing a metallophore activity toward the rare earth elements that does not appear to be related to ionic radii or previously reported EC_50_ concentrations for *E. coli*. We found the strength of metallophore activity towards these elements was as follows: Pr > Sc > Eu > Tm > Tb > Er > Yb > Ce > Lu > Sm > Ho > La > Nd > Dy > Gd > Y. This is the first study to investigate such activity and indicates bacteria may provide a means of removal/recovery of these critical elements.

## 1. Introduction

The study of bacteria has been an ongoing process for hundreds of years. While the field itself covers a broad area of specialties, one field of study that has been gaining significant interest is that of microbe–metal interactions [1,2,3,4,5,6]. While studies have been carried out related to the direct impact of oxyanions of metals and metalloids on bacteria, a significantly lower amount of attention has been placed on the ability of certain microbes to ‘collect’ such metal ions via secreted proteins [7,8,9,10,11,12,13]. Many bacteria possess low-weight molecules (<2000 Da) called siderophores [14], which are essential to their survival. The job of a siderophore is to collect iron (Fe) from the environment to be brought back to the cell when it is lacking this essential element in its immediate surroundings, thus allowing for survival under low-Fe conditions. However, while the main purpose of siderophores is Fe acquisition, they appear to have additional roles, one of which is the binding of other elements, such as copper (Cu) and zinc (Zn) rather than Fe [12,15]. In some cases, the affinity for Cu or Zn can actually be higher than that for Fe [16,17]. Therefore, the term ‘metallophore’ has been coined to refer to these secondary metabolites that have an affinity for various different metals [18]. This idea of multiple metal(loid) capture has been implicated in tolerance to toxic heavy metal(loid) oxyanions [12], particularly in extreme environments. In this case, the toxicity of the metal is related to its ability to enter the cell. Many metal ions can freely diffuse through the cell membrane unless they are bound to a metallophore. This means that if a metal is bound to this molecule, it will be rejected from entering the cell, thereby reducing the toxicity. The idea of reducing the toxicity of these compounds by immobilizing them through binding to a metallophore is an interesting one and would aid our understanding of metal(loid) resistance/tolerance among various bacteria. This theory is supported by the fact that many bacteria which possess extremely high-level resistance to certain metals and/or metalloids do in fact possess metallophores with the ability to bind the metal to which they are resistant. For example, while *Blastomonas ursincola* KR99 is known for its tellurite resistance (MIC 2000 µm/mL K_2_TeO_3_) [19], it is also known to have very strong metallophore activity toward tellurium (Te) [12]. As the main method of toxicity for Te oxyanions involves the creation of radical oxygen species inside the cell due to its strong oxidative properties [20], the strong binding ability of this bacterium’s secreted metallophore may contribute to its impressive resistance. The same can be said regarding selenite resistance, as bacteria known to possess high levels of tolerance also possess metallophore activity towards selenium (Se) [12,19].

In addition to this aspect, other possible benefits may arise, such as bioremediation and biomining. Many environments have been contaminated by metals and bacteria have been gaining significant attention as a potentially cost effective ‘green’ method of remediation. Work has been done showing that these microbes do have the ability to remove toxic metal(loid)s from their surroundings, but the breadth of metal(loid)s that can be removed is still under investigation. Recent works have indicated many bacteria do in fact possess metallophores for several metals, including nickel (Ni), Cu, cobalt (Co), manganese (Mn), Zn, vanadium (V), and several others [12]. This indicates the potential for metallophores being produced against numerous elements. As one can see, bacterial metallophore activity towards a wide range of metals has been studied, but one such group that has yet to receive much attention in this regard is the rare earth elements (REEs). These are a group of 17 elements which include 15 lanthanides typically categorized as light (Lanthanum (La), Cerium (Ce), Praseodymium (Pr), Neodymium (Nd), Promethium (Pm) Samarium (Sm), and Europium (Eu)) and heavy (Gadolinium (Gd), Terbium (Tb), Dysprosium (Dy), Holmium (Ho), Erbium (Er), Thulium (Tm), Ytterbium (Yb), and Lutetium (Lu)), along with yttrium (Y) and scandium (Sc) (due to their similar chemical properties) [21]. As Pm is radioactive, it is typically not considered when discussing uses of the REEs and is not considered in this work. While this group is referred to as ‘rare’, in reality most are not [22]. For example, Ce is the 25th most abundant element on Earth, which is greater than more ‘common’ elements such as lead (Pb) and tin (Sn). These elements have gathered substantial interest in recent years due to their unique characteristics, which give them unique catalytic, magnetic, and electronic properties [22]. Due to these features, REEs are used in a wide array of products. La has been commonly used in batteries associated with electric vehicles, along with lesser amounts of Ce, Nd, and Pr [23]. La and Sm have a role in superconductors [24], with Er and Y borides being in solar panels [25]. Beyond these more specialty items, REEs are also found in non-electric vehicles (mainly Ce in catalytic converters) [26] and are commonly used in metallurgical processes [23]. Beyond industrial applications, they also have important functions in the medical field [27]. Gd, Ho, Tm, Nd, and Er have a role in medical imaging devices, due to their electron density. For example, Gd is used as a contrast agent in magnetic resonance imaging (MRI) [28].

In the case of toxicity of the REEs, they do have limiting effects on bacteria. For example, CeO_2_ nanoparticles can be toxic to bacteria, such as *E. coli* and *Bacillus subtilis*, at as low as 290 µM [29]; and Dy_2_O_3_ at 5.3 µM inhibits *E. coli* [30]. Findings such as these suggest the rare earth elements may be significantly dangerous to microbes; however, other work has indicated this may not be the case. Certain studies have suggested that the toxicity of certain REEs is actually not as severe (Tm: ~1000 µM, La: ~800 µM) [31]. In regard to microbial community structure, the presence of these metals appears to have a negative impact. It appears that the presence of REEs exerts a sustained inhibition on both microbial abundance and community composition in soils impacted by in situ leaching mining [32]. With results such as these, it would appear that in reality the true toxic effect of REEs on bacteria is still somewhat of a mystery.

Due to this great demand for REEs, obtaining them has become a lucrative business. Many REEs are somewhat omnipresent, but the concentrations are too low to be recovered by traditional means in an economical manner [21,33]. This, coupled to the current increased necessity for these compounds, lends itself to finding a new approach to obtain them. If specific biological proteins can be discovered that bind such elements, and therefore could potentially concentrate/collect them, this could lead to a cost-effective, environmentally friendly method for acquiring the REEs using bacteria, something which is gaining interest [34,35]. This biomining approach has received attention, with the major hurdle being identifying microorganisms which can target desired metal(loid)s. Work has been done showing that microbes do have the ability to remove/sequester REEs from their surroundings [36,37], but our knowledge of the breadth of this activity is severely lacking. Subsequently, as our use of these elements increases, products containing them will inevitably be discarded, leading to increased concentrations locally. Therefore, fully understanding the impact of the REEs on microbes is of paramount importance.

In this study we set forth to determine if bacteria possessing siderophore activity would also have metallophore activity towards the REEs. Such a question has yet to be investigated and would be a significant leap forward in our understanding of bacterial interaction with this group of elements. To that end, we identified eight bacterial strains, six isolated from sediment of acid mine drainage from coal mining and two from a natural reference site that possessed siderophore activity. They were then screened for metallophore activity against all 16 nonradioactive REEs to determine if such an ability does in fact exist. In regard to bioreclamation of the REEs, the discovery of bacteria which produce an external metal binding protein is exciting.

## 2. Materials and Methods

### 2.1. Environmental Sampling Locations and Siderophore Screening

Three locations were chosen for sampling, two of which were impacted by acid mine drainage (AMD) from coal mining, and one natural site as a reference. The first site chosen was an untreated AMD stream at the Jennings site (before remediation treatment) [38]. This location is a stream that is fed with water directly coming from an abandoned coal mine. No attempt at treatment has been undertaken at this sampling location. The second is an AMD treated stream, Slippery Rock Elementary Wetland [39]. Here, an artificial wetland was created to aid in the remediation of drainage from coal mining. While the location has undergone treatment, the remnants of mine drainage still impact the site. Finally, there is a natural site, Wolf Creek [40]. This was used as the reference site as this waterway has never been exposed or impacted by mine drainage. Sediment samples were taken from each of the three sites and a 10-fold serial dilution was carried out (*w*/*v*) through 10^−5^. The pH of sediment from the sites was as follows: Jennings, 6.2; Slippery Rock Elementary Wetland, 6.8; Wolf Creek, 7.2. Each sample dilution was plated on Rich Organic (RO) [41] agar pH 7.0 at 30 °C in the dark for 24 h. These plates were supplemented with the dye Chromeazurol S (CAS), which, when bound to a metal ion, is blue in color. If that metal ion is removed/released from the dye molecule, the color changes to yellow/orange [42]. To make this media for monitoring siderophore activity, 60.5 mg CAS was dissolved in 50 mL ddH_2_O, then mixed with 10 mL of a metal solution containing 1 mM of Fe^3+^ and 10 mM HCl. Then, 72.9 mg of Hexadecyltrimethylammonium bromide (HDTMA) was dissolved in 40 mL ddH_2_O prior to mixing with the CAS/metal solution, bringing the total volume to 100 mL. Media was then autoclaved at pH 5.9, separate from the CAS mixture. Following this, each solution was adjusted to pH 6.8 with 0.5 N NaOH and the CAS solution was added [12]. Agar plates turn blue when solidified if made correctly. If the pH were incorrect, or components added in the wrong order, the plates would appear green instead of blue.

### 2.2. Rare Earth Element Metallophore Screening

Due to the chemical properties of CAS, one can replace Fe^3+^ for cations of the rare earth elements. The eight strains identified with siderophore activity (ES1, ES2, ES3, ES4, J1, J2, WC1, and WC2) were used for metallophore activity screening. The isolates were plated on RO agar plates supplemented with CAS solution made with the corresponding chloride salt of the desired rare earth element using the same concentration as Fe^3+^ for siderophore screening (10 mL of a 1 mM solution), resulting in CAS plates containing Cerium (Ce^3+^), Dysprosium (Dy^3+^), Erbium (Er^3+^), Europium (Eu^3+^), Gadolinium (Gd^3+^), Holmium (Ho^3+^), Lanthanum (La^3+^), Lutetium (Lu^3+^), Neodymium (Nd^3+^), Praseodymium (Pr^3+^), Samarium (Sm^3+^), Scandium (Sc^3+^), Terbium (Tb^3+^), Thulium (Tm^3+^), Ytterbium (Yb^3+^), and Yttrium (Y^3+^). The only REE not used was Promethium (Pm), as it is radioactive. Following solidification, all plates were various shades of blue, depending on the metal used, indicating they were properly made. Each strain was inoculated onto a CAS plate containing one of the REEs and then incubated for 24 h at 30 °C. Afterwards, plates were observed for a color change from blue to yellow/orange around bacterial growth, indicating removal of the rare earth element cation from the CAS dye as a result of metallophore activity. The distance from the edge of growth to the edge of color change (zone of activity) was recorded in mm and used to determine relative strength of metallophore activity (Figure S1). All experiments were performed in triplicate.

### 2.3. 16S rRNA Gene Sequencing

The cells of each isolate were subjected to DNA extraction as described in [43] and sent to Azenta (Burlington, MA, USA) for Sanger sequencing of the complete 16S rRNA gene to determine their phylogenetic positions. Gene sequences of each isolate can be found in GenBank under the corresponding accession numbers (Table S1).

## 3. Results

### 3.1. Siderophore Activity

**Figure 1:**
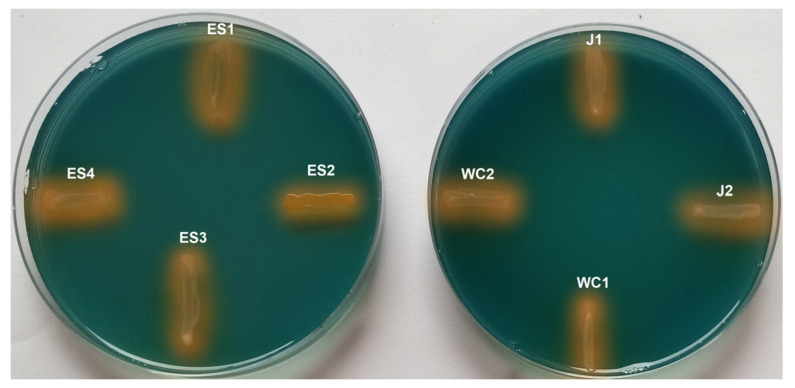
Growth on a CAS plate coupled with siderophore activity for each of the eight isolates identified in this study. Activity is represented by a change in media color from blue to yellow/orange.

Following incubation, each dilution plated on CAS plates containing Fe^3+^ was observed for any colonies surrounded by a yellow/orange coloration, indicating siderophore activity. Growth on these plates was limited, and siderophore activity appeared to be relatively rare. The proportions of colonies from each site with activity were as follows: Jennings (17.6%), Slippery Rock Elementary Wetland (15.3%, and Wolf Creek (11.6%). We found a total of eight unique isolates that displayed this coloration (ES1, ES2, ES3, ES4, J1, J2, WC1, and WC2). These were selected and re-screened for siderophore activity individually on a CAS Fe^3+^ plate to confirm results. As well as confirming siderophore production, this step also indicated that all selected strains could grow properly on a CAS plate, as this is not always the case. The presence of siderophores was confirmed in all eight isolates (Figure 1), indicating they could be used for further metallophore screening.

### 3.2. Rare Earth Element Metallophore Activity

**Figure 2:**
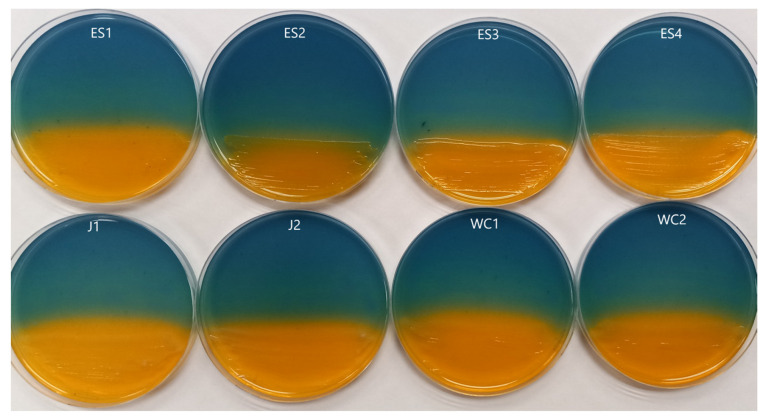
Metallophore activity of each isolate on CAS plates containing Samarium. Color change from bluish to yellow/orange indicates release of Sm^3+^ from the dye as a result of metallophore activity. Similar results for all other rare earth elements.

**Figure 3:**
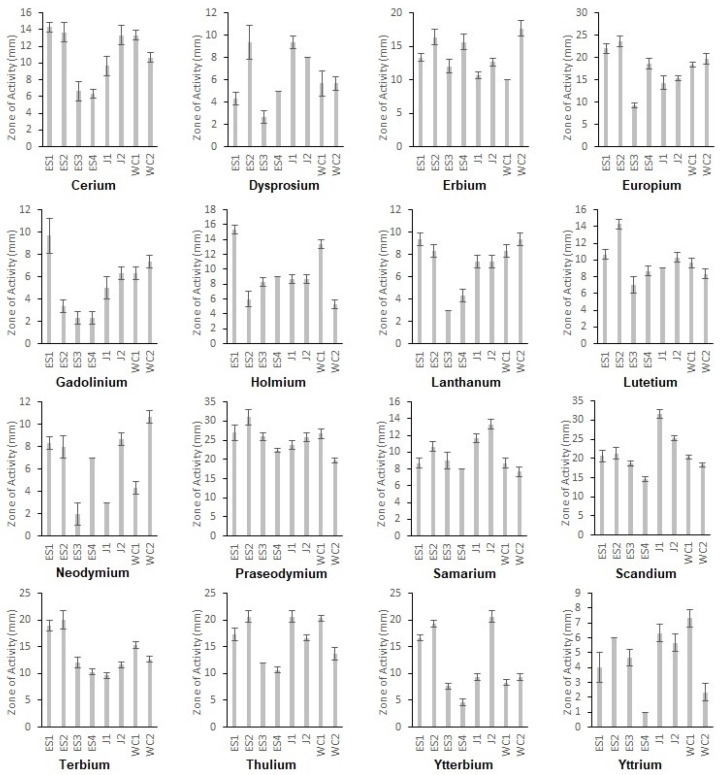
Average size of the Zone of Activity (in mm) for all eight isolates towards each rare earth element.

Each isolate that produced a siderophore and could grow on a CAS plate was then used to inoculate a subsequent RO CAS plate containing one of the 16 rare earth elements in place of Fe^3+^. After incubation, we found all eight isolates possessed metallophore activity against each of the rare earth elements used. This was indicated by a yellow/orange coloration surrounding the growth (Figure 2 and Figure S2). While each strain had activity toward each rare earth element, possibly indicating a similar method of action, not all activity was of the same strength (Figure 3 and Figure S2, Table 1 and Table S2). Strains ES1, ES2, J1, J2, WC1, and WC2 had zones of activity ranging from 11.15 to 14.50 mm on average across all rare earth elements, while strains ES3 and ES4 were only 8.96–9.29 mm on average (Table S2). In addition, not all rare earth elements had the same strength of metallophore activity. For example, Pr had a zone of activity of up to 31 mm for strain ES2, while Y had only a 1 mm zone of activity for strain ES4. Overall, the strengths of metallophore activity against all tested rare earth elements were as follows:

**Pr > Sc > Eu > Tm > Tb > Er > Yb > Ce > Lu > Sm > Ho > La > Nd > Dy > Gd > Y**

### 3.3. 16S rRNA Gene Sequencing

Based on sequencing, one can see that the majority of isolates belong to Pseudomonas (Table 2). This genus is well known for its interactions with metals [44], so this result is not surprising. The other genera represented are not well known for their interactions with metals and may represent novel abilities.

## 4. Discussion

Bacterial interactions with metals in regard to metallophore activity are just beginning to be explored. Previous work has shown that many strains with siderophore activity also possess binding activity against a wide range of metals [12,18]. In addition, many isolates inhabit extreme environments, including acid mine drainage. In line with these previous studies, we found isolates with siderophore activity in sites impacted by mining. The communities at Jennings and Slippery Rock Elementary Wetland had the most siderophore-producing strains at 17.6% and 15.3%, respectively, with the natural site, Wolf Creek, having the lowest proportion at 11.6%. It may seem counterintuitive to have siderophore production in an iron-rich environment; however, as we found, the proteins produced by these strains are not limited to iron. Based on our findings, metallophore activity extends to the rare earth elements as well, since all eight isolates possess activity against all 16 rare earth elements tested. Additionally, of the eight isolates studied here, six came from sites impacted by acid mine drainage due to coal mines, suggesting that environments such as these, which are typically rich in metals, may be a prime location for these abilities to evolve. The strength of metallophore activity did vary significantly, suggesting that some of these metals may indeed be exerting some type of toxic effect. It is known that certain rare earth elements are known to be more toxic than others; however, this does not fully explain our results. For example, one study indicated that Lu and Y are the most toxic REEs to most organisms, while Ce and Nd are the least toxic [45]. Other studies show similar findings for most toxicity, but put Sc as the most toxic, behind Lu and Y, and suggest that Pr and Tb are the least toxic [46]. Our work actually supports the idea of Y being the most toxic (least metallophore activity) but shows Lu as intermediate and Sc as having the second strongest activity. Conversely, our findings support the idea of Pr being the least toxic (strongest metallophore activity) but show Tb and Ce in the middle range of activity and Nd with the fourth lowest activity. Another theory to the impact of REEs on bacteria is linked to their sizes [45], noting that decreasing toxicity followed increasing radii. When applied to our findings for metallophore activity, it does not seem to be directly correlated. The smallest element (Sc), based on ionic radii [45], has the second most activity, while the largest (La) has the fifth lowest activity. In addition, if you look at all the rare earth elements, no pattern appears based on ionic radii, with some of the larger REEs having lower activity and some of the smaller higher activity (Table 3). Prior work with *Pseudomonas fluorescens,* in regard to interactions with REEs, has suggested a link between a decrease in adsorption of these elements with increasing atomic number [47]; however, our findings do not follow this pattern either. Overall, it would appear that many variables are in play when determining metallophore activity and toxicity, and we have just begun to scratch the surface as to these interactions.

Lastly, it should be noted that there are limited studies on the true toxicity of the rare earth elements to bacteria, with only one published study investigating the toxicity of all REEs, focusing on *E. coli* [46], a less than ideal model for strains with high resistance to metals. Based on sequencing, one can see that the majority of these isolates belong to *Pseudomonas* (Table 2). Studies have suggested that this genus may have a role to play in bioleaching of REEs. *P. aeruginosa* has shown promise to leach these elements from monazite [49]. *P. fluorescens* can adsorb these metals as well [47]. In regard to the other genera identified in this study (*Serratia, Yersinia,* and *Chryseobacterium*), metal interactions are known. *Serratia marcescens* is known to have high-level resistance to certain metals [50,51], *Yersinia pestis* is known to have metallophores, some similar to those from *P. aeruginosa* [52], and *Chryseobacterium* species can also resist metals [53] but are not known for metallophore production. One study has implicated a siderophore in *Pseudomonas fluorescence* for binding of various REEs (La, Ce, Pr, Nd, Sm, Eu, Gd, Tb, Dy, Ho, and Er) [47]. Perhaps something similar is happening here, as all our isolates have siderophore activity. Finding metallophore activity raises the question of why bacteria might possess this ability. Bacteria that use lanthanides in the active sites of methanol dehydrogenases have been identified [36]. In the case of the family *Beijerinckiaceae*, they rely on REEs for methanol oxidation [54]. For them, methanol oxidation is only possible in the presence of La, Ce, and Nd at submicromolar concentrations. The question as to how these bacteria acquire these elements is still unknown, but perhaps they are employing some type of metallophore, which is logical based on our findings. In addition to this, there are bacteria that possess not only lanthanide-bearing cofactors but also other proteins with a high affinity and selectivity for other REEs. One example is lanmodulin, which, upon binding to lanthanides, undergoes a conformational change and has a 100-million-fold higher affinity for these elements compared to calcium [55]. Another finding in *Pseudomonas putida* KT2440 is that La, Ce, Pr, Sm, and Nd have direct roles in the activity of alcohol dehydrogenases, as well as transcriptional regulation of these genes [56].

A second implication of metal-binding proteins is in the field of biomining. Increased demand, coupled with expensive, difficult acquisition and sparse sources, is driving the search for a more effective and efficient method of mining/recovery. It is known that wastewater contains these elements, but the concentrations are extremely low and current methods of treatment ignore this resource. Biofilms have been investigated in this regard as a means to aggregate these elements and recover them from wastewater [57]. Another approach is the use of phosphate-solubilizing bacteria to leach the rare earth elements from ore [58]. These studies, along with many others, are highlighting the amazing potential of bacteria in recovery/recycling of these important elements. Possibly, metallophore compounds will have a place in the future as they are tailored to bind these metals and are naturally secreted from the cell, meaning that they could be produced en masse, ‘released’, and then somehow ‘collected’, now bound to a highly valuable element. The actual implication of a method such as this would require a significant amount of further study, but our work here may provide a steppingstone towards this end. As demand for the REEs increases, bacteria may have a role to play in acquiring these sparse elements through the use of metallophore compounds, which would be a step forward based on our current mining methods. Abilities to interact with REEs may be novel among their respective groups. It would appear that in terms of interactions with and the toxicity of this group of metals, more work is required to determine the true impacts on bacteria across many different genera.

## 5. Conclusions

In conclusion, investigations into the interaction of bacteria with REEs need to continue. As we are using an increased amount of these elements in everyday devices, such as solar panels, batteries, and electric cars, we are by default introducing them into the environment at increased levels. This leads to greater interactions with microbes, and without fully understanding their impact we may be causing long term issues of which we are unaware. Our work has shown that bacteria do in fact have methods of directly interacting with REEs that may be related to siderophore activity. This is the first study of its kind and the first to identify metallophores which interact with REEs. As discussed, the majority of our isolates were members of *Pseudomonas*, a genus known to possess siderophores and metallophores. While we have discovered metallophore activity, the actual properties of the compound responsible for this remain unknown and one can only speculate on how it may function. In addition, the true levels of toxicity of these elements have yet to be studied beyond *E. coli*. In the end, one can see that what we know about REEs and bacteria needs to be greatly expanded. In order to do this, we need a better understanding of how bacteria interact with and are affected by the metals. For this, a more suitable model system is required. The typical use of *E. coli*, as used in prior studies, is not reasonable as it does not possess the abilities or resistance of the other species discussed here. We suggest that, based on this work and prior studies, a better model system for investigation would use a *Pseudomonas* species. As we have discussed, they can utilize rare earth elements in biochemical and physiological processes and also possess metallophores capable of binding these metals.

## Figures and Tables

**Table 1:** Strength of metallophore activity against the rare earth elements.

Strain	Ce	Dy	Er	Eu	Gd	Ho	La	Lu	Nd	Pr	Sm	Sc	Tb	Tm	Yb	Y
ES1	++	+	++	+++	++	++	++	++	++	++++	++	+++	+++	+++	+++	+
ES2	++	++	++	+++	+	++	++	++	++	++++	++	+++	+++	+++	+++	++
ES3	++	+	++	++	+	++	+	++	+	+++	++	+++	++	+++	++	+
ES4	++	+	++	+++	+	++	+	++	++	+++	++	++	++	+++	+	+
J1	++	++	++	++	+	++	++	++	+	+++	++	+++	++	+++	++	++
J2	++	++	++	++	++	++	++	++	++	+++	++	+++	++	+++	+++	++
WC1	++	+	++	+++	++	++	++	++	+	++++	++	+++	++	+++	++	++
WC2	++	++	+++	+++	++	+	++	++	++	+++	++	+++	++	++	++	+

+ = 1–5 mm Zone of Activity; ++ = 6–15 mm; +++ = 16–25 mm; ++++ = >25 mm.

**Table 2:** Isolation source and nearest relatives of each isolate based on 16S rRNA gene sequencing. All strains were found in sediment samples from their respective sites.

Strain	Nearest Relative	Isolation Source
ES1	*Pseudomonas oryziphila* (99.93%)	Slippery Rock Elementary Wetland
ES2	*Chryseobacterium vietnamense* (98.99%)	Slippery Rock Elementary Wetland
ES3	*Pseudomonas rhizophila* (99.48%)	Slippery Rock Elementary Wetland
ES4	*Pseudomonas frederiksbergensis* (99.70%)	Slippery Rock Elementary Wetland
J1	*Yersinia entomophaga* (99.63%)	Jennings
J2	*Serratia fonticola* (99.41%)	Jennings
WC1	*Pseudomonas mosselii* (99.80%)	Wolf Creek
WC2	*Pseudomonas hamedanensis* (99.63%)	Wolf Creek

**Table 3:** Ionic radii of each rare earth element (in the ^3+^ state) as compared to the strength of metallophore activity and their toxicity to *E. coli*.

Rare Earth Element	Ionic Radii (Å) *	Average Zone of Metallophore Activity (mm)	*E. coli* EC_50_ During Exponential Phase (µM) ^#^
Scandium (Sc)	0.87	21.38	1.10
Lutetium (Lu)	0.98	9.75	3.00
Ytterbium (Yb)	0.99	12.00	3.98
Thulium (Tm)	0.99	16.50	6.30
Erbium (Er)	1.00	13.54	8.30
Holmium (Ho)	1.02	9.33	17.40
Yttrium (Y)	1.02	4.67	7.60
Dysprosium (Dy)	1.03	6.25	18.60
Terbium (Tb)	1.04	13.83	21.90
Gadolinium (Gd)	1.05	5.33	17.20
Europium (Eu)	1.07	17.67	20.50
Samarium (Sm)	1.08	9.71	17.20
Neodymium (Nd)	1.11	6.50	19.40
Praseodymium (Pr)	1.13	25.25	27.40
Cerium (Ce)	1.14	11.00	19.70
Lanthanum (La)	1.16	7.17	18.10

* [48]; ^#^ [46].

## Data Availability

Not Applicable.

## Supplementary Materials

The following supporting information can be downloaded at: https://www.mdpi.com/article/10.3390/microorganisms11112672/s1, Figure S1: Measurement of the Zone of Activity for metallophores taken from the edge of growth to the edge of color change due to metallophore activity; Figure S2: Range of metallophore activity against the rare earth elements for each bacterial isolate. Activity is seen based on the media changing from bluish to yellow/orange around growth due to metallophore activity; Table S1: GenBank accession number for the 16S rRNA gene sequences of the 8 isolates used in this study; Table S2: Zone of Activity of metallophores for each isolate and average Zone of Activity for each rare earth element in mm.
