# Supplementary material for: Metallophore Activity toward the Rare Earth Elements by Bacteria Isolated from Acid Mine Drainage Due to Coal Mining
Source: Microorganisms. 2023 Oct 31;11(11):2672. doi: 10.3390/microorganisms11112672 (PMC10673398; doi:10.3390/microorganisms11112672)
Supplement: Supplementary file 1 [file microorganisms-11-02672-s001.zip › microorganisms-2664159-supplementary.pdf]

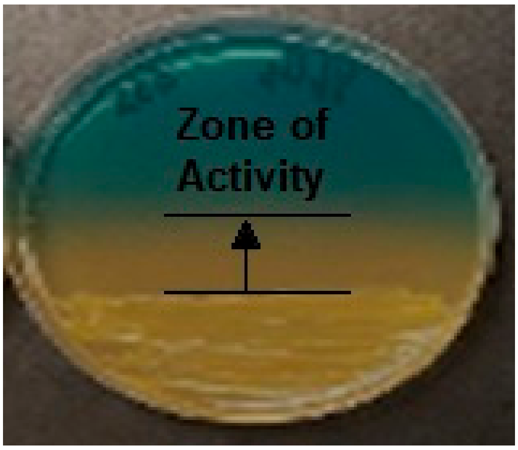

Figure S1. Measurement of the Zone of Activity for metallophores taken from the edge of growth to the edge of color change due to metallophore activity.

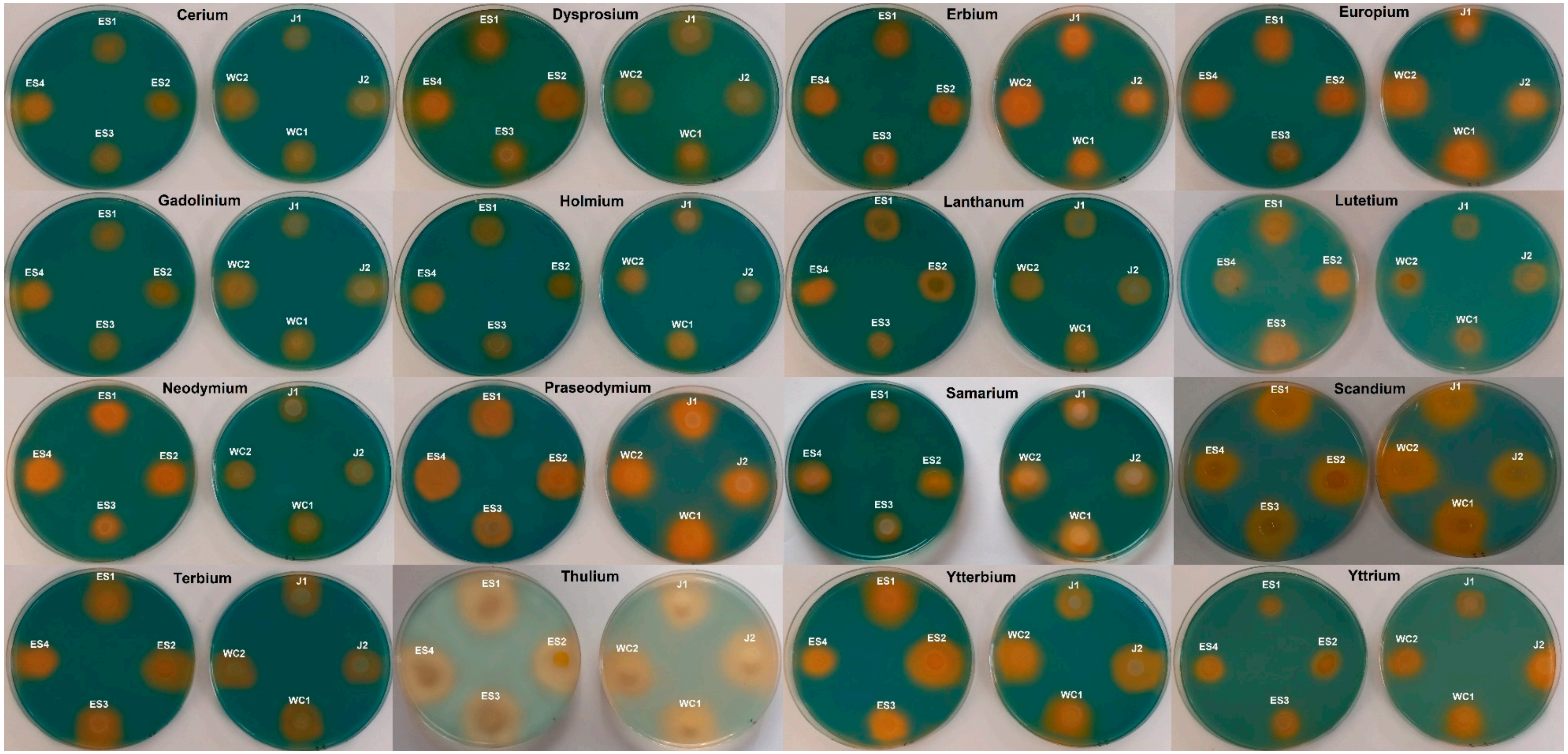

Figure S2. Range of metallophore activity against the rare earth elements for each bacterial isolate. Activity is seen based on the media changing from bluish to yellow/orange around growth due to metallophore activity.

Table S1. GenBank accession number for the 16S rRNA gene sequences of the 8 isolates used in this study.

Strain	Accession Number
ES1	OR496588
ES2	OR496587
ES3	OR520911
ES4	OR496592
J1	OR496585
J2	OR496589
WC1	OR496400
WC2	OR496583

Table S2. Zone of Activity of metallophores for each isolate and average Zone of Activity for each REE in mm.

Element	ES1	ES2	ES3	ES4	J1	J2	WC1	WC2	Average
**Ce**	14.33±0.58	13.67±1.15	6.67±1.15	6.33±0.58	9.67±1.15	13.33±1.15	13.33±0.58	10.67±0.58	11.00±3.20
**Dy**	4.33±0.58	9.33±1.53	2.67±0.58	5.00±0.00	9.33±0.58	8.00±0.00	5.67±1.15	5.67±0.58	6.25±2.42
**Er**	13.33±0.58	16.33±1.15	12.00±1.00	15.67±1.15	10.67±0.58	12.67±0.58	10.00±0.00	17.67±1.15	13.54±2.76
**Eu**	22.00±1.00	23.67±1.15	9.33±0.58	18.67±1.15	14.33±1.53	15.33±0.58	18.33±0.58	19.67±1.15	17.67±4.57
**Gd**	9.67±1.53	3.33±0.58	2.33±0.58	2.33±0.58	5.00±1.00	6.33±0.58	6.33±0.58	7.33±0.58	5.33±2.59
**Ho**	15.33±0.58	6.00±1.00	8.33±0.58	9.00±0.00	8.67±0.58	8.67±0.58	13.33±0.58	5.33±0.58	9.33±3.40
**La**	9.33±0.58	8.33±0.58	3.00±0.00	4.33±0.58	7.33±0.58	7.33±0.58	8.33±0.58	9.33±0.58	7.17±2.32
**Lu**	10.67±0.58	14.33±0.58	7.00±1.00	8.67±0.58	9.00±0.00	10.33±0.58	9.67±0.58	8.33±0.58	9.75±2.19
**Nd**	8.33±0.58	8.00±1.00	2.00±1.00	7.00±0.00	3.00±0.00	8.67±0.58	4.33±0.58	10.67±0.58	6.50±3.05
**Pr**	27.00±2.00	31.00±2.00	26.00±1.00	22.33±0.58	23.67±1.15	25.67±1.15	26.67±1.15	19.67±0.58	25.25±3.40
**Sc**	20.67±1.53	21.33±1.53	18.67±0.58	14.67±0.58	31.67±1.15	25.33±0.58	20.33±0.58	18.33±0.58	21.38±5.14
**Sm**	8.67±0.58	10.67±0.58	9.00±1.00	8.00±0.00	11.67±0.58	13.33±0.58	8.67±0.58	7.67±0.58	9.71±1.99
**Tb**	19.00±1.00	20.00±1.73	12.00±1.00	10.33±0.58	9.67±0.58	11.67±0.58	15.33±0.58	12.67±0.58	13.83±3.89
**Tm**	17.33±1.15	20.67±1.15	12.00±0.00	10.67±0.58	20.67±1.15	16.67±0.58	20.33±0.58	13.67±1.15	16.50±4.01
**Yb**	16.67±0.58	19.33±0.58	7.67±0.58	4.67±0.58	9.33±0.58	20.67±1.15	8.33±0.58	9.33±0.58	12.00±5.99
**Y**	4.00±1.00	6.00±0.00	4.67±0.58	1.00±0.00	6.33±0.58	5.67±0.58	7.33±0.58	2.33±0.58	4.67±2.14
**Average**	13.79±6.47	14.50±7.70	8.96±6.37	9.29±5.93	11.88±7.46	13.10±6.28	12.27±6.37	11.15±5.36	
